# Performance of a Logistic Regression Model Using Paired miRNAs to Stratify Abnormal Mammograms for Benign Breast Lesions

**DOI:** 10.1002/cam4.70767

**Published:** 2025-04-15

**Authors:** Hideo Akiyama, Lora Barke, Therese B. Bevers, Suzanne J. Rose, Jennifer J. Hu, Kelly A. McAleese, Shellie S. Campos, Satoshi Kondou, Jun Atsumi, Thomas F. Soriano

**Affiliations:** ^1^ Toray Industries, Inc. Kamakura Kanagawa Japan; ^2^ Invision Sally Jobe/Radiology Imaging Associates Englewood Colorado USA; ^3^ Division of OVP, Department of Clinical Cancer Prevention, Cancer Prevention and Population Sciences The University of Texas MD Anderson Cancer Center Houston Texas USA; ^4^ Department of Research and Discovery, Stamford Health, Breast Center Stamford Health Stamford Connecticut USA; ^5^ Department of Public Health Science University of Miami School of Medicine Miami Florida USA; ^6^ The Women's Imaging Center Denver Colorado USA; ^7^ John Muir Health, Walnut Creek and Concord California USA; ^8^ Toray Industries, Inc. Tokyo Japan; ^9^ Diagnostic Oncology CRO, LLC Oxford Connecticut USA

**Keywords:** abnormal mammogram, benign breast lesions, breast cancer, liquid biopsy, serum miRNA

## Abstract

**Background:**

Mammography is effective in reducing breast cancer mortality, but it has false positive results that cause subsequent interventions such as biopsy or interval repeat mammography. Thus, there is a clinical unmet need for accurate molecular classifiers that can reduce unnecessary additional imaging and/or invasive diagnostic procedures for low‐risk women.

**Method:**

We performed miRNA profiling on a prospectively collected serum specimen obtained from each of the 432 subjects who received an abnormal mammogram or imaging result and then selected 265 subjects for further analysis. The miRNA classifier, named EarlyGuard, was generated based on a novel logistic regression model using “paired miRNAs” where the two miRNAs of interest exhibit the same properties.

**Results:**

The classifier developed using the training set of 174 subjects enrolled at seven investigative sites resulted in a negative predictive value (NPV) and a sensitivity of 96.4% and 91.2%, respectively. The classifier was validated using the test set consisting of 91 subjects enrolled at three investigative sites, two of which were not included in the training set. The resulting NPV and sensitivity were estimated similarly to be 96.9% and 95.8%, respectively.

**Conclusions:**

Our miRNA classifier has produced promising results that could be used in conjunction with mammography or other imaging procedures to reduce unnecessary invasive diagnostic procedures for women who are unlikely to have a suspicious or worse result on a subsequent diagnostic biopsy. Additional studies will be conducted in larger cohorts to determine if the sensitivity of the classifier will be improved.

## Abstract 2

We have generated EarlyGuard, a novel logistic regression model, using five paired miRNAs, to stratify subjects with abnormal mammograms who are proven benign, that is, neither breast cancer nor a pre‐cancerous diagnosis, on a subsequent diagnostic biopsy at an NPV of 96.9%. The classifier could be used as a “rule‐out” test in the management of subjects with suspicious breast imaging findings who undergo subsequent biopsy of the breast and would have a diagnosis of neither breast cancer nor a pre‐cancerous condition (e.g., ductal carcinoma in situ [DCIS]).

## Graphical abstract

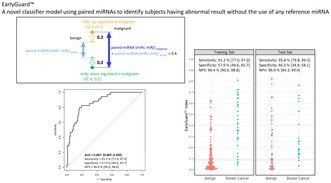

## 1 Introduction

Mammography is considered the principal non‐invasive imaging method of screening for breast cancer worldwide. It has been shown to be effective in reducing breast cancer deaths in several randomized studies [1, 2]. However, screening mammography can result in false positives that cause subsequent interventions such as biopsy or interval repeat mammography. It is estimated that each year $2.8 billion is spent in the U.S. as a result of false‐positive mammograms in women 40–59 years of age [3]. Furthermore, up to one third of cancers detected by mammography screening would never have resulted in clinical symptoms during the woman's lifetime [4]. False positive results also have the consequence of increased short‐term anxiety in women who undergo unnecessary diagnostic biopsies [5]. While attempts have been made to develop new imaging techniques such as MRI, ultrasound, and others to improve breast cancer screening, none has yet been shown to reduce breast cancer death rates to the degree that mammograms have [6]. It is important to note that the benefits of mammography screening, such as early detection of breast cancer, reduction in breast cancer mortality, and morbidity of breast cancer treatment, need to be balanced against its harms, which include overdiagnosis and anxiety.

Recently, multiple advanced imaging methods and non‐invasive biomarkers, including circulating microRNAs (miRNAs), have been developed to improve breast cancer detection [7, 8, 9]. miRNAs are single‐stranded non‐coding RNAs with 19–25 nucleotides [10], and circulating miRNAs have been cited as promising minimally invasive markers for breast cancer [11, 12]. However, circulating miRNAs that are dysregulated in benign breast lesions can vary depending on the type of lesion and the individual. Therefore, it is important to study miRNA expression patterns in a large number of subjects with malignant and benign lesions of the breast to identify common dysregulated miRNAs that have diagnostic or therapeutic potential in blood.

Furthermore, an important challenge in the clinical translation of circulating miRNA biomarkers is the selection of an appropriate miRNA data normalization process to reduce technical variation and allow accurate comparison and analysis of results [13, 14, 15, 16, 17]. While the double delta cycle threshold (Ct) method [18] is a well‐known approach for the analysis of circulating miRNA data, there is no current consensus about an optimal normalization strategy for miRNA quantification. Therefore, the selection of a reliable reference miRNA in liquid biopsy studies requires several important criteria, such as stability in the biological conditions and reproducibility of technical and biological biases.

In this study, we aimed to develop a novel miRNA classifier without the use of any reference miRNA specifically to aid in determining the likelihood that a subject with a suspicious breast imaging finding will not have breast cancer or a pre‐cancerous diagnosis on a subsequent diagnostic biopsy.

## 2 Methods

### 2.1 Subject Enrollment

This study was approved by the following Institutional Review Boards (IRBs), Covenant HealthCare [Covenant Medical Center IRB: C‐18‐33 TRY‐003D], Invision Sally Jobe [HCA—HealthONE IRB: 1321554], John Muir Medical Imaging [John Muir Health IRB: HRP‐402], MD Anderson Cancer Center [MDACC Office of Human Subject Protection: PA‐19‐0033], Overlake Hospital [WCG IRB: 1248949], Scottsdale Medical Imaging [WCG IRB: 1249910], Stamford Health [WCG IRB: 1250516], University of Miami [University of Miami Human Subject Research Office (M809): 20190042], Women's Imaging Center [WCG IRB: 1252754], and Toray Industries Inc. [Human Tissue Samples Ethics Committee for R&D: HC2020‐13, HC2021‐13, HC2022‐13, HC2023‐02006]. The written informed consent was obtained prior to the scheduling of women for the blood draw. Whole blood specimens were collected prospectively under the IRB‐approved protocol from women who received suspicious breast imaging results (Breast Imaging Reporting and Data System (BI‐RADS) Assessment Category of 4 (Suspicious Abnormality) or 5 (Highly Suggestive of Malignancy)) that had been read by a radiologist trained in breast imaging (obtained from diagnostic mammography, ultrasound, and/or MRI) and who underwent a standard of care diagnostic biopsy within 60 days to determine the pathological characteristics. The available pathology slides and breast images were re‐examined by one or two additional expert pathologists and radiologists for adjudication, respectively.

### 2.2 Serum Collection and Processing

The whole blood specimens were collected and processed at each institution using red/gray topped gel barrier tubes (Becton Dickinson Vacutainer SST Tubes 367,988, Franklin Lakes, NJ). The blood specimens were allowed to clot for 30–35 min at room temperature. The tubes were then centrifuged at either 4°C or room temperature at 1100–1300 g for 10 min. After centrifugation, aliquots of 0.5 mL of serum were stored at or below −80°C within 90 min of collection. Each clinical institution used a color chart available from the Centers for Disease Control (CDC) (https://www.cdc.gov/vector‐borne‐diseases/php/laboratories/reference‐tool‐for‐hemolysis‐status.html) to check for the presence of hemolysis above a hemoglobin level of 50 mg/dL, as hemolysis can indicate the destruction of red blood cells, which could affect the miRNAs found in the serum.

### 2.3 Non‐Cancer Healthy Female Serum Collection and Processing

The whole blood specimens were collected by Discovery Life Sciences (Huntsville, AL) and processed as described above from five individual non‐cancer healthy female donors on a biweekly basis, for a total of six collections per donor.

### 2.4 miRNA Microarray Analysis

Total RNA was extracted from 0.3 mL of serum using the *3D‐Gene* RNA extraction reagent (Toray Industries Inc., Tokyo, JAPAN), according to the manufacturer's protocol. A comprehensive miRNA microarray analysis was performed at 42°C for 17 h using the *3D‐Gene* Human miRNA Oligo Chip v.22 (Toray Industries Inc.) and the corresponding miRNA signal was evaluated according to the manufacturer's instructions. The miRNA microarray data with fewer than 250 miRNA signals were excluded from further analysis. To identify reliable miRNAs, only those found to be expressed in 100% of the specimens in the training set were selected.

### 2.5 Linearity of miRNA Signal Intensity

The whole blood specimens were collected from women enrolled in this study. Total RNA was extracted from 0.3 mL of each serum as described above. Four serial two‐fold (2×) dilutions of each serum RNA solution were used for *3D‐* *Gene* miRNA microarray analysis in triplicate to determine a linear relationship, as the linear coefficient of determination and the slope of the regression line between the concentration and the Log_2_ signal intensity of each miRNA.

### 2.6 Statistical Analysis

The datasets of miRNA signals as described above were divided into training and test sets for the model development and performance evaluation of the miRNA classifier. In the model development, a logistic regression model was created using the training set with paired miRNA that is the following Log_2_ ratio of two miRNAs of interest, miR_*i*_ and miR_*j*_, as explanatory variables.

$$\text{Paired miRNA}\;\left({\mathrm{miR}}_{i},{\mathrm{miR}}_{j}\right)=\log_{2}{\text{signal}}_{{\mathrm{miR}}_{i}}-\log_{2}{\text{signal}}_{{\mathrm{miR}}_{j}}=\log_{2}\frac{{\text{signal}}_{{\mathrm{miR}}_{i}}}{{\text{signal}}_{{\mathrm{miR}}_{j}}}$$

**FIGURE 1:**
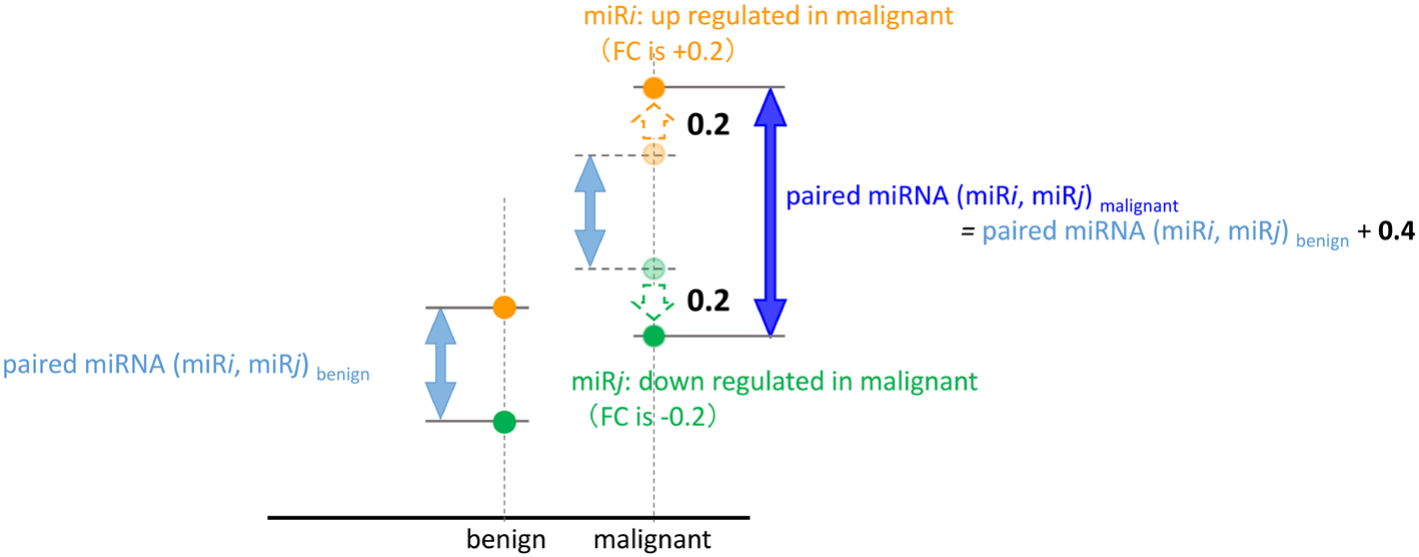
Concept of a logistic regression model using paired miRNAs. The Log_2_ ratio of two miRNAs of interest, miR_*i*_, miR_*j*_, is expressed as follows: $\text{Paired miRNA}\;\left({\mathrm{miR}}_{i},{\mathrm{miR}}_{j}\right)=\log_{2}{\text{signal}}_{{\mathrm{miR}}_{i}}-\log_{2}{\text{signal}}_{{\mathrm{miR}}_{j}}$. In case that the two miRNA, miR_*i*_ and miR_*j*_, are up‐ and downregulated respectively by 0.2 in malignant breast lesions, the paired miRNA in malignant lesions as paired miRNA (miR_*i*_, miR_*j*_)_malignant_ is greater than that in benign lesions, the paired miRNA (miR_*i*_, miR_*j*_)_benign_, by 0.4. The fold‐change ratio (FC) is used as a quantitative indicator and is obtained without the use of any reference miRNAs as normalizers, thus removing any normalization bias.

For paired miRNA to be a suitable explanatory variable, it is required that the extraction and hybridization efficiencies as well as stability of miR_*i*_ and miR_*j*_ are approximately equal and that both signals increase in proportion to the concentration of the miRNAs showing similar slopes. Theoretically, in the case that miR_*i*_ and miR_*j*_ are up‐ and down‐regulated, respectively, by 0.2 in malignant breast lesions, the Log_2_ ratio of two miRNAs (miR_*i*_, miR_*j*_) in malignant lesions, paired miRNA (miR_*i*_, miR_*j*_) _malignant_, will be greater than that in benign lesions by 0.4 (Figure 1).

Classification models were created using up to a maximum of five paired miRNAs from all paired miRNAs that satisfied the above conditions. Limiting the number of paired miRNAs to five ensures the robustness and reliability of the logistic regression models, maintains quality control and reproducibility for diagnostic applications, and helps to manage computational resources effectively while still providing a robust classification model. Classification was optimized by the Best Subset Selection Logistic model using the sequential primal‐dual active set (SPDAS) algorithm [19] based on AIC [20] (BeSS package version 2.0.3 in R software version 4.0.5 [21]). A cut‐off value used to classify neither breast cancer nor a pre‐cancerous diagnosis on a subsequent diagnostic biopsy by the predicted probability of the logistic regression model was determined through the highest specificity with a sensitivity of at least 90% in the training set. The 95% confidence interval (CI) was calculated using the Wilson score and the DeLong methods for proportions and the area under the curve (AUC) of a receiver‐operator characteristic (ROC) curve (pROC package version 1.17.0.1 [22]), respectively. Since the Log_2_‐transformed signal intensity of the miRNA represents a normal distribution, we assume that the resulting Log_2_ ratio of the paired miRNA could also represent a normal distribution. Therefore, the differences in the Log_2_ ratio of the paired miRNAs between benign and malignant lesions were assessed using a two‐tailed *t*‐test.

The correlation analysis between the classifier and age was assessed using the Pearson correlation coefficient and a two‐tailed *t*‐test on the slope of the regression line to determine if it was significantly different from zero. On the other hand, the correlations between the classifier and clinical covariates, including BI‐RADS category, breast composition category, and race/ethnicity, were evaluated using a one‐way analysis of variance (one‐way ANOVA) for groups containing at least two subjects in each category and visualized using violin plots.

The influence of the time of blood draw on the classifier was assessed using correlation analysis of a set of both the Log_2_ signal intensities of the miRNAs and the Log_2_ ratio of the paired miRNAs between each time point, as the index was generated with the paired miRNAs.

## 3 Results

### 3.1 Performance Evaluation of the Logistic Regression Model Using Paired miRNAs on Serum Specimens

We hypothesized that an accurate miRNA‐based logistic regression model development would require an algorithm trained on real‐world specimens collected from women who received suspicious breast imaging results (BI‐RADS Category 4 or 5), followed by a standard of care diagnostic biopsy. To achieve this, we conducted a prospective clinical study in which a single blood specimen was collected from each enrolled woman prior to a screening breast imaging procedure at nine geographically distributed sites, encompassing both prevalent and rare breast cancer as well as various benign lesions. Adjudication was confirmed by experts on both the tissue assessment reported for the breast imaging studies and the pathology diagnoses on the breast biopsy tissues and the pathology categorization of the disease, as shown in Table 1.

**TABLE 1:** Pathology categories.

Benign	Suspicious/atypical	Other (in situ)	Breast cancer
Fibroadenoma	Atypical ductal hyperplasia (ADH)	Ductal carcinoma in situ (DCIS; when highest lesion)	Invasive carcinoma (of any type)
Fibrosis	Atypical lobular hyperplasia (ALH)		Mucinous carcinoma
Fibrocystic changes (proliferative or nonproliferative)	Lobular carcinoma in situ (LCIS)		Phyllodes, malignant^a^
(Fibro) adipose	Atypical (micropapillary) intraductal proliferation		DCIS with microinvasion
(Micro) cysts	Phyllodes, borderline^a^		
Papillary apocrine metaplasia			
Usual ductal hyperplasia (UDH)			
Flat epithelial atypia (FEA)^b^ (without atypia)			
Intraductal papilloma (without atypia)			
Pseudoangiomatous stromal hyperplasia (PASH)^c^			
Radial sclerosing lesion/radial scar			
Phyllodes, benign^a^			

*Note:* The pathology diagnoses on the breast biopsy tissues from women who received suspicious breast imaging results (BI‐RADS 4 or 5) were categorized as “Other”, “Suspicious/atypical”, “Benign”, or “Cancer”.

^a^ Phyllodes is assessed based on the recognized subtypes: benign, borderline, and malignant.

^b^ Flat epithelial atypia (FEA) without atypia is classified as benign. FEA with atypia is categorized based on the highest lesion present (i.e., ADH—suspicious/atypical; DCIS—other category).

^c^ PASH is classified as benign because studies have demonstrated that patients with PASH do not have a subsequent increased risk of cancer.

Comprehensive miRNA expression analysis with four serial 2× dilutions of serum RNA solution was performed in triplicate as described above. The reliable signal of each miRNA, which is the directly proportional relationship between the amount of serum RNA and the corresponding Log_2_ signal intensity, was calculated. The 201 miRNAs to be “paired miRNAs” were selected based on the criteria for the linear coefficient of determination (≥ 0.975) between the concentration and signal intensity of miRNA detected in serum RNA.

**FIGURE 2:**
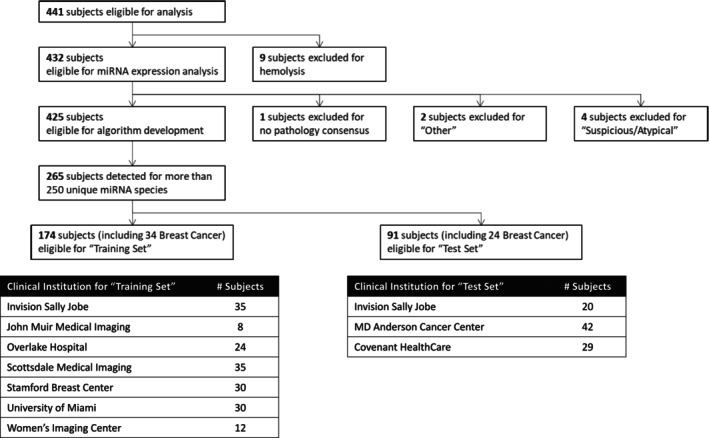
Study flowchart. Number of subjects included and excluded in this study. Subjects were excluded according to the stated criteria. Each clinical institution used a color chart to check for the presence of hemolysis above a hemoglobin level of 50 mg/dL as hemolysis can indicate the destruction of red blood cells which could affect the miRNAs found in the serum.

**FIGURE 3:**
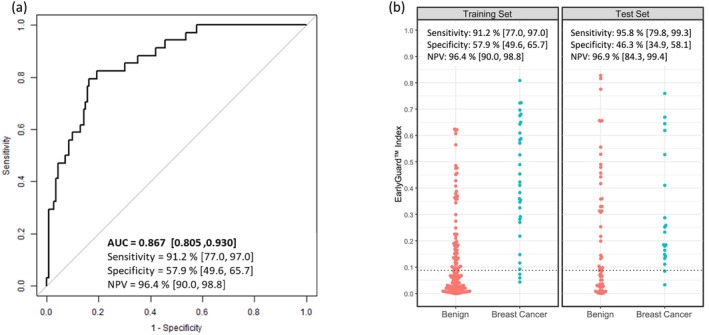
Performance of EarlyGuard classifier. (a) ROC curve for the performance of the EarlyGuard classifier in the training set comprising 34 malignant and 140 benign lesions. The AUC, sensitivity, specificity, and negative predictive value (NPV) were shown. Each 95% CI was calculated using the Wilson score and the DeLong methods for proportions and the AUC of a ROC curve, respectively and shown in brackets. (b) Beeswarm plot for performance of the EarlyGuard classifier in the training (34 malignant and 140 benign lesions) and test (24 malignant and 67 benign lesions) sets. The sensitivity, specificity, and NPV were shown.

**FIGURE 4:**
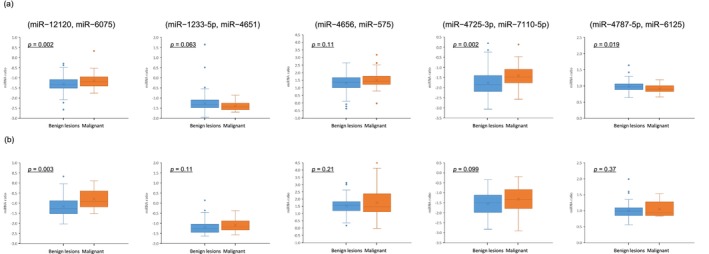
EarlyGuard classifier comprising five paired miRNA in malignant and benign breast lesions. (a) Boxplots of the Log_2_ ratio of each paired miRNA between malignant and benign lesions in the training set (34 malignant and 140 benign lesions). (b) Boxplots of Log_2_ ratio of each paired miRNA between malignant and benign lesions in the test set (24 malignant and 67 benign lesions). Significant differences in both the training and test sets were observed in paired miRNA (miR‐12120, miR‐6075) and (miR‐4725‐3p, miR‐7110‐5p).

Among 441 subjects enrolled in this study, sixteen (16) subjects were excluded from the algorithm development due to hemolysis of the specimen, lack of pathology consensus, or categorization as “Other” or “Suspicious/Atypical.” These exclusions were made to avoid bias in data analysis or misleading analysis outcomes. miRNA expression data were obtained from a total of 425 prospectively enrolled subjects. Of these, 160 subjects (37 malignant and 123 benign) with fewer than 250 miRNA signals were excluded from the further analysis based on the assumption that the data with insufficient RNA yield could introduce bias into the algorithm development, thereby enhancing the reliability and robustness of the classifier. The remaining subjects were randomly divided into training (*n* = 174) and test (*n* = 91) sets at the clinical institution level without regard to clinical characteristics or miRNA signal information, as shown in Figure 2. The 174 subjects (34 malignant and 140 benign) were used to develop a classifier that could stratify a subject with a suspicious breast imaging finding as not having breast cancer or a pre‐cancerous diagnosis on a subsequent diagnostic biopsy. Only 179 miRNAs present in 100% of the specimens were considered for further analysis to ensure the robustness and reliability of our results. All possible paired miRNAs of these 179 miRNAs were assessed, resulting in 3623 combinations of two miRNAs as paired miRNAs where the slopes of the regression line were within 0.05 of each other. A logistic regression model was then optimized using these combinations, which led to the generation of the EarlyGuard classifier index = 1/(1 + Exp^−*x*^), *x* = 4.16 × (miR‐12120, miR‐6075) − 3.82 × (miR‐1233‐5p, miR‐4651) + 1.63 × (miR‐4656, miR‐575) + 1.93 × (miR‐4725‐3p, miR‐7110‐5p) − 6.30 × (miR‐4787‐5p, miR‐6125) + 5.19. The area under the curve (AUC) of a receiver‐operator‐characteristic (ROC) curve of the training set was 0.867 (95% confidence interval (CI): 0.805–0.930) and with an optimal cut‐off value of 0.0892 for the EarlyGuard index, the negative predictive value (NPV), sensitivity, and specificity were 96.4% (95% CI: 90.0%–98.8%), 91.2% (95% CI: 77.0%–97.0%), and 57.9% (95% CI: 49.6%–65.7%), respectively (Figure 3). In other words, the index ≥ 0.0892 indicates “malignant,” whereas the index < 0.0892 indicates “neither cancer nor a pre‐cancerous diagnosis on a subsequent diagnostic biopsy.” The performance of the classifier was consistent when validated in the test set of 91 prospectively enrolled subjects from three clinical institutions, two of which were not included in the training set, with NPV of 96.9% (95% CI: 84.3%–99.4%), sensitivity of 95.8% (95% CI: 79.8%–99.3%), and specificity of 46.3% (95% CI: 34.9%–58.1%) (Figure 3). The Log_2_ ratio of each of the five paired miRNAs obtained from the training set was similar to that from the test set (Figure 4). Any outliers observed in Figure 4 were not excluded from statistical analyses between the malignant and benign lesions cohorts in the training and test sets as the corresponding Log_2_ signal intensity of each miRNA was evaluated by the scan images according to the manufacturer's instructions, indicating that the outliers should be of biological significance rather than technical variation. Therefore, the differences in two paired miRNAs, (miR‐12120, miR‐6075) and (miR‐4725‐3p, 7110‐5p), were statistically and biologically significant between the malignant and benign breast lesions and consistently observed in both the training and test sets without any reference RNAs used for data normalization.

### 3.2 Study Subject Characteristics

The pathological and clinicopathological characteristics of the subject cohorts eligible for the training and test sets are shown in Tables 2 and 3. The training and test sets for the benign breast lesions cohort constituted a heterogeneous group of lesions arising in the mammary epithelium or other mammary tissues, and benign lesions with more than one histological pathology were classified with the highest pathological result. As shown in Table 2, the Chi‐squared test reveals that the pathological characteristics of the benign breast lesions cohort were significantly different between the training and test sets (*p* < 0.001).

**TABLE 2:** Pathological characteristics of malignant and benign breast lesions used for the training (*N* = 174) and test (*N* = 91) sets.

Subtype	Training set (*N* = 174)	Test set (*N* = 91)
Total benign, *n* (%)	140	67
Chi‐squared test: *p* < 0.001		
ibroadenoma	57 (40.7)	14 (20.9)
Fibrosis	8 (5.7)	7 (10.4)
Fibrocystic changes	42 (30.0)	19 (28.4)
(Fibro) adipose	11 (7.9)	5 (7.5)
(Micro) cysts	4 (2.9)	0 (0)
Papillary apocrine metaplasia	6 (4.3)	1 (1.5)
Usual ductal hyperplasia (UDH)	5 (3.6)	10 (14.9)
Flat epithelial atypia (FEA)	0 (0)	0 (0)
Intraductal papilloma	2 (1.4)	7 (10.4)
PASH	1 (0.7)	2 (3.0)
Radial sclerosing lesion/radial scar	4 (2.9)	2 (3.0)
Phyllodes, benign	0 (0)	0 (0)
Total malignant, *n* (%)	34	24
Chi‐squared test: *p* = 0.32		
Invasive carcinoma (of any type)	32 (94.1)	23 (95.8)
Mucinous carcinoma	1 (2.9)	1 (4.2)
Phyllodes, malignant	1 (2.9)	0 (0)
DCIS with microinvasion	0 (0)	0 (0)

*Note:* Chi‐squared tests were performed to assess differences in the clinical characteristic of malignant and benign breast lesions between the training and test sets.

**TABLE 3:** Clinicopathological characteristics of subject cohorts for the training (*N* = 174) and test (*N* = 91) sets.

Malignant	Training set (*N* = 34)	Test set (*N* = 24)
Age	[*t*‐test: *p* = 0.46]	
Mean	55.2	57.5
Median	53.5	57.5
Range	33–80	40–76
Race/ethnicity, *n* (%)	[Chi‐squared test: *p* = 0.78]	
Caucasian, non‐Hispanic	27 (79.4)	20 (83.3)
Caucasian, Hispanic	3 (8.8)	3 (12.5)
Asian	2 (5.9)	0 (0)
African American	0 (0)	0 (0)
Black Hispanic	1 (2.9)	0 (0)
Other	1 (2.9)	1 (4.2)
BI‐RADS assessment category, *n* (%)	[Chi‐squared test: *p* = 0.42]	
BI‐RADS 2	0 (0)	0 (0)
BI‐RADS 3	0 (0)	0 (0)
BI‐RADS 4	14 (41.2)	10 (41.7)
BI‐RADS 5	17 (50.0)	13 (54.2)
Other	3 (8.8)	1 (4.2)
Breast composition category, *n* (%)	[Chi‐squared test: *p* = 0.41]	
A	0 (0)	1 (4.2)
B	10 (29.4)	6 (25.0)
C	18 (52.9)	16 (66.7)
D	1 (2.9)	0 (0)
Unknown	5 (14.7)	1 (4.2)
Receptor status, *n* (%)	[ER: *p* = 0.033, PR: *p* = 0.034, HER2: *p* = 0.19]	
ER, positive	28 (82.5)	21 (87.5)
ER, negative	5 (14.7)	3 (12.5)
ER, unknown	1 (2.9)	0 (0)
PR, positive	27 (79.4)	19 (79.2)
PR, negative	5 (14.7)	5 (20.8)
PR, unknown	2 (5.9)	0 (0)
HER2, positive	3 (8.8)	2 (8.3)
HER2, negative	26 (76.5)	22 (91.7)
HER2, unknown	5 (14.7)	0 (0)
Invasive carcinoma, *n* (%)	[Chi‐squared test: *p* = 0.049]	
Grade 1	8 (23.5)	8 (33.3)
Grade 2	16 (47.1)	9 (37.5)
Grade 3	9 (26.5)	7 (29.2)
Unknown	1 (2.9)	0 (0)

*Note:* A two‐tailed *t*‐test was performed to assess differences in age of malignant and benign breast lesions between the training and test sets. Chi‐squared tests were performed to assess differences in other clinicopathological characteristics, race/ethnicity, BI‐RADS assessment categories, receptor status, and invasive carcinoma of malignant and benign breast lesions between the training and test sets.

Regarding the clinicopathological characteristics of the subject cohorts, the training and test sets include subjects of Caucasian Non‐Hispanic, Caucasian Hispanic, Asian, African American, and Other races/ethnicities. The median ages of the subjects in the malignant lesions cohort in the training and test sets were 53.5 (ranged 33–80 years) and 57.5 (ranged 40–76 years) years, respectively, and there was no significant difference between the training and test sets. For the malignant diagnoses cohort, both the training and test sets had similar proportions of BI‐RADS Assessment Categories, BI‐RADS Breast Tissue Assessment groupings, receptor status, and tumor grade based on the results of the Chi‐squared tests. However, each clinicopathological characteristic of the benign breast lesions cohort was significantly different between the training and test sets, indicating the diversity of the benign breast lesions cohort. The probability of cancer among subjects in the training and test sets was 19.5% and 26.4%, respectively. In contrast, the probability of cancer among the subjects excluded from the algorithm development was 23.1%, indicating that the subject diversity was not affected by the selection of eligible subjects.

### 3.3 Validation of the EarlyGuard Index

**FIGURE 5:**
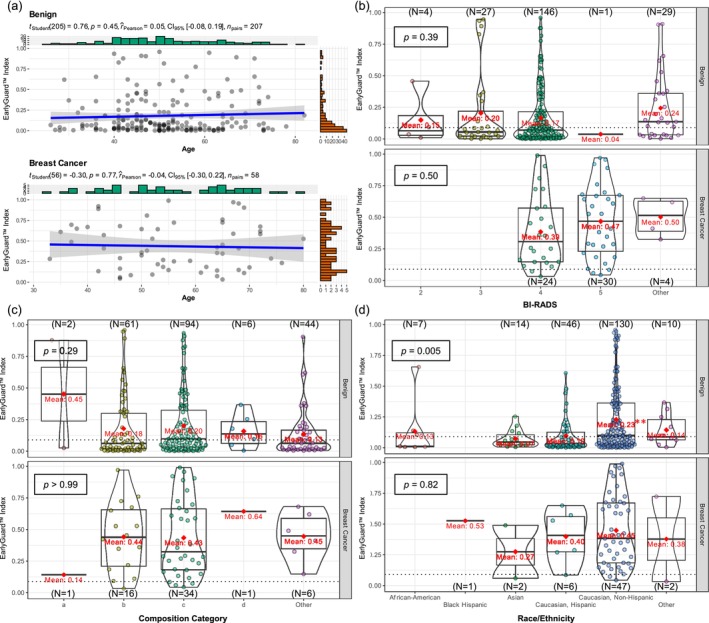
Correlation and violin‐box plots of EarlyGuard classifier and clinical covariates. (a) Correlation analysis between ages of the subject and EarlyGuard index in the benign (upper panel) and breast cancer (lower panel) cohorts. The gray circles represent each subject in the benign or breast cancer cohorts. The *p*‐values for the slopes of the regression lines in the benign and malignant cohorts were 0.45 and 0.77, respectively. Pearson correlation for benign and malignant cohorts were 0.05 and −0.04, respectively. (b) Violin‐box plots for BI‐RADS category of EarlyGuard index in the benign (upper panel) and breast cancer (lower panel) cohorts. The circles in pink, yellow, green, blue, and purple represent each subject in BI‐RADS category 2, 3, 4, 5, and Other, respectively. The red rhombuses represent the mean indices, which were consistent across categories in each cohort, indicating that BI‐RADS category was not associated with the EarlyGuard index. A higher mean index was observed in the malignant cohort. The *p*‐values of the one‐way ANOVA in the benign and malignant breast lesions cohorts were 0.39 and 0.50, respectively. The dotted line indicated the cut‐off score of 0.0892 for the EarlyGuard index. (c) Violin‐box plots for breast composition category of EarlyGuard index in the benign (upper panel) and breast cancer (lower panel) cohorts. The circles in pink, yellow, green, blue, and purple represent each subject in breast composition category a, b, c, d, and Other. The red rhombuses represent the mean indices. A similar higher mean index as in BI‐RADS categories was observed in the malignant cohort. The *p*‐values of the one‐way ANOVA in the benign and malignant breast lesions cohorts were 0.29 and > 0.99, respectively. The dotted line indicates the cut‐off score of 0.0892 for the EarlyGuard index. (d) Violin‐box plots for race/ethnicity of EarlyGuard index in the benign (upper panel) and breast cancer (lower panel) cohorts. The circles in pink, yellow, green, pale blue, blue, and purple represent each subject in race/ethnicity—African‐American, Black Hispanic, Asian, Caucasian‐Hispanic, Caucasian‐Non Hispanic, and Other, respectively. The red rhombuses represent the mean indices. The *p*‐values of the one‐way ANOVA in the benign and malignant breast lesions cohorts were 0.005 and0.82, respectively. A trend was observed in Caucasian non‐Hispanics with slightly higher indexes compared to Caucasian, Hispanics, and Asians in both benign (upper panel) and breast cancer (lower panel) cohorts.

There is no information available on how significant variables including age, BI‐RADS category, breast composition category, and race/ethnicity affect the resulting EarlyGuard classifier indexes. The relationship between age and the EarlyGuard index was assessed using a *t*‐test to determine whether the slope of the regression line was significantly different from zero. As shown in Figure 5a, the correlation analysis demonstrates that the ages of the subjects in the benign (*n* = 207) and malignant (*n* = 58) breast lesions cohorts were not associated with a likelihood of the EarlyGuard index, as the *p*‐values for the slopes of the regression lines in the malignant and benign subject cohorts were 0.45 and 0.77, respectively. Additionally, the Pearson correlation coefficient for malignant and benign cohorts was 0.05 (95% CI: −0.08, 0.19) and −0.04 (95% CI: −0.30, 0.22), respectively, indicating that there was no significant association between age and the EarlyGuard index in both benign and malignant breast lesions cohorts.

Based on the violin‐box plots for BI‐RADS category, a similar distribution of the index was observed across each BI‐RADS category but differed between the malignant and benign subject cohorts, resulting in a higher mean index in the malignant cohort (Figure 5b). However, the mean indices were consistent across categories in each cohort as the *p*‐values of the one‐way ANOVA in the benign and malignant breast lesions cohorts were 0.39 and 0.50, respectively, indicating that BI‐RADS category was not associated with the EarlyGuard index. A similar distribution pattern of the index in BI‐RADS categories was observed in the breast composition categories (Figure 5c). On the other hand, a trend was observed in both malignant and benign cohorts of Caucasian non‐Hispanics, with slightly higher indices compared to Caucasian Hispanics and Asians (Figure 5d). The *p*‐values of the one‐way ANOVA in the benign and malignant breast lesions cohorts were 0.005 and 0.82, respectively, suggesting that the EarlyGuard index of Caucasian non‐Hispanics in the benign lesions cohort would be significantly different among other races/ethnicities. Future studies will be necessary to investigate the potential impact of race/ethnicity on the performance of this classifier as the number of subjects in each race/ethnicity category was uneven and some categories had very few subjects enrolled.

### 3.4 Influence of Timing of Blood Draw on the EarlyGuard Index

**FIGURE 6:**
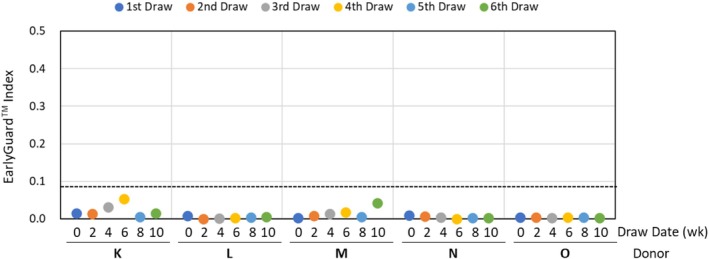
Influence of pre‐analytical factors on EarlyGuard classifier indexes. The subjects were mostly drawn in the early afternoon each day that they had a blood specimen collected. The EarlyGuard indices were obtained from Log_2_ signal values of the 10 miRNAs without the use of reference or control miRNA for data normalization. The dashed line indicates the cut‐off score of 0.0892 for the EarlyGuard index. In other words, an index ≥ 0.0892 indicates “malignant,” whereas an index < 0.0892 indicates “neither cancer nor a pre‐cancerous diagnosis on a subsequent diagnostic biopsy.” The resulting EarlyGuard classifier indices of five individual non‐cancer healthy female donors were stable over six time points and stratified as subjects without breast cancer or without pre‐cancerous diagnosis on a subsequent diagnostic biopsy.

Serum miRNA levels have been known to be affected by specimen processing conditions, such as the time after blood draw, storage conditions, centrifugation conditions, time after centrifugation, and circadian changes. Therefore, blood specimens were drawn from five non‐cancer healthy female donors on a bi‐weekly basis, for a total of six collections per donor to assess the influence of the frequency of blood drawn on the EarlyGuard index. The Pearson correlation coefficients of the correlation matrix of the signal intensities of 10 miRNA and the resulting five paired miRNAs over six time points varied from 0.983 to 0.999 and 0.953 to 0.999, respectively, indicating that the EarlyGuard 10 miRNA expression profiles among six time points per donor are reproducible and highly correlated (Table 4). The indices of five individual non‐cancer healthy female donors were stable over six time points during ten consecutive weeks and classified as “neither cancer nor a pre‐cancerous diagnosis on a subsequent diagnostic biopsy” (Figure 6). This suggests that the EarlyGuard index potentially discriminates between women who have a malignant diagnosis on subsequent biopsy and those women who had benign breast lesions or apparently healthy women, regardless of the timing of the blood collection.

**TABLE 4:** Correlation matrix of EarlyGuard classifier.

	Panel (a)					
	K_1st	K_2nd	K_3rd	K_4th	K_5th	K_6th
Donor K						
K_1st	1					
K_2nd	0.994	1				
K_3rd	0.996	0.998	1			
K_4th	0.998	0.997	0.998	1		
K_5th	0.997	0.997	0.999	0.998	1	
K_6th	0.998	0.993	0.995	0.996	0.997	1

	L_1st	L_2nd	L_3rd	L_4th	L_5th	L_6th
Donor L						
L_1st	1					
L_2nd	0.995	1				
L_3rd	0.998	0.998	1			
L_4th	0.998	0.995	0.999	1		
L_5th	0.997	0.998	0.997	0.997	1	
L_6th	0.999	0.995	0.996	0.997	0.996	1

	M_1st	M_2nd	M_3rd	M_4th	M_5th	M_6th
Donor M						
M_1st	1					
M_2nd	0.994	1				
M_3rd	0.992	0.999	1			
M_4th	0.997	0.998	0.996	1		
M_5th	0.996	0.998	0.998	0.997	1	
M_6th	0.99	0.999	0.999	0.996	0.996	1

	N_1st	N_2nd	N_3rd	N_4th	N_5th	N_6th
Donor N						
N_1st	1					
N_2nd	0.996	1				
N_3rd	0.996	0.996	1			
N_4th	0.989	0.983	0.991	1		
N_5th	0.998	0.996	0.997	0.993	1	
N_6th	0.999	0.997	0.995	0.99	0.999	1

	O_1st	O_2nd	O_3rd	O_4th	O_5th	O_6th
Donor O						
O_1st	1					
O_2nd	0.997	1				
O_3rd	0.996	0.998	1			
O_4th	0.998	0.996	0.999	1		
O_5th	0.997	0.998	0.999	0.998	1	
O_6th	0.998	0.997	0.999	0.999	1	1

	Panel (b)					
	K_1st	K_2nd	K_3rd	K_4th	K_5th	K_6th
Donor K						
K_1st	1					
K_2nd	0.999	1				
K_3rd	0.989	0.981	1			
K_4th	0.998	0.996	0.991	1		
K_5th	0.99	0.982	0.99	0.983	1	
K_6th	0.99	0.984	0.979	0.982	0.997	1

	L_1st	L_2nd	L_3rd	L_4th	L_5th	L_6th
Donor L						
L_1st	1					
L_2nd	0.985	1				
L_3rd	0.993	0.99	1			
L_4th	0.996	0.985	0.998	1		
L_5th	0.982	0.993	0.979	0.98	1	
L_6th	0.997	0.994	0.995	0.994	0.985	1

	M_1st	M_2nd	M_3rd	M_4th	M_5th	M_6th
Donor M						
M_1st	1					
M_2nd	0.964	1				
M_3rd	0.958	0.996	1			
M_4th	0.996	0.979	0.973	1		
M_5th	0.985	0.99	0.981	0.989	1	
M_6th	0.953	0.99	0.997	0.971	0.969	1

	N_1st	N_2nd	N_3rd	N_4th	N_5th	N_6th
Donor N						
N_1st	1					
N_2nd	0.987	1				
N_3rd	0.98	0.996	1			
N_4th	0.985	0.995	0.989	1		
N_5th	0.991	0.987	0.992	0.982	1	
N_6th	0.998	0.992	0.988	0.993	0.994	1

	O_1st	O_2nd	O_3rd	O_4th	O_5th	O_6th
Donor O						
O_1st	1					
O_2nd	0.997	1				
O_3rd	0.982	0.993	1			
O_4th	0.986	0.995	0.999	1		
O_5th	0.991	0.998	0.997	0.997	1	
O_6th	0.989	0.997	0.999	0.999	1	1

*Note:* Panel (a) Correlation matrix of the EarlyGuard 10 miRNA expression profile in healthy individuals over six time points during ten consecutive weeks. Panel (b) Correlation matrix of the EarlyGuard 5 paired miRNAs in healthy individuals over six time points during ten consecutive weeks.

## 4 Discussion

The false positive result in the breast cancer screening process is a significant concern to both patients and health care providers. An estimated 5%–12% of prevalently screened women were recalled for a second procedure, and some had received further procedures [23], resulting in increased short‐term anxiety and inconvenience for the women undergoing the second procedure, including diagnostic biopsy of the breast and increased costs to the health care system. The logistic regression model comprising five paired miRNAs, EarlyGuard, was optimized for differentiating malignant and benign breast lesions among women with BI‐RADS Category 4 or 5 mammogram results enrolled at the nine investigative sites. In addition, the results of the classifier are not associated with significant variables such as age, BI‐RADS category, breast composition category, and race/ethnicity, suggesting that the EarlyGuard index has the potential to dramatically improve the care and outcomes of women who have a BI‐RADS Category 4 or 5 mammography result.

Among the ten miRNAs in our logistic regression model, miR‐575 has been known as an oncogene in many tumors by targeting CDKN1B and BRCA1 [24]. Moreover, miR‐575 is associated with the development of gastric cancer by targeting PTEN, known as a tumor suppressor gene, at the transcription level [25, 26, 27]. Interestingly, miR‐575 in serum was reported to be upregulated in breast, colon, and lung cancer patients [28]. Furthermore, miR‐1233‐5p in serum has been shown to be downregulated in breast cancer patients who responded to nivolumab [29]. It has also been shown that miR‐4656 regulates the proliferation of breast cancer cell lines by targeting CSNK2B at the transcription level [30, 31].

Furthermore, miR‐6075 in serum has been shown to be elevated in patients with lung cancers as well as in those with pancreatic and biliary tract cancer [32, 33]. For miR‐4651, it has been reported to repress cell growth, proliferation, and migration by targeting FOXP4 and BRD4 in liver and lung tumor tissues, respectively [34, 35]. Interestingly, FOXP4 is expressed highly in breast tumor tissues compared to adjacent normal tissues, and its upregulation is associated positively with many clinicopathologic factors, such as tumor size, pathological grade, and metastasis [36]. Also, BRD4 is a well‐known transcriptional regulator that plays a critical role in promoting breast cancer cell proliferation, survival, malignancy, and migration [37]. Similarly, miR‐6125 has been shown to downregulate YTHDF2 and inhibit the growth of colorectal cancer cells by downregulating cyclin D1 [38]. Specifically, cyclin D1 has been shown to be involved in driving breast cancer initiation and progression by contributing to ERα activation [39]. The overexpression of cyclin D1 has been inversely associated with tumor grade and positively associated with the ER and PR status in invasive ductal carcinoma [40]. Taken together with previous findings, the identification of miR‐575 and miR‐6125 correlations through cyclin D1/CDK in ER‐positive breast cancer proliferation may facilitate the development of predictive biomarkers and novel therapeutic targets. Apart from the functions of the miRNAs, two selected circulating miRNAs might be suitable for use as an explanatory variable. Since the two miRNAs of each miRNA pair could share the same properties in terms of their extraction and hybridization efficiency, stability, and quantification, the paired miRNAs can remove differences due to input and quality of RNA and can identify true changes in miRNA expression between serum collected from subjects with malignant and benign breast lesions.

There are other significant miRNA biomarker candidates and models for the classification of abnormal mammograms for breast cancer that have been published. A miRNA signature (miR‐451a, miR‐195‐5p, miR‐126‐5p, miR‐423‐3p, miR‐192‐5p, and miR‐17‐5p) measured in serum has been reported to stratify malignant breast lesions in women with abnormal screening mammograms at an AUC of 0.774 in a validation cohort with NPV of > 80% [12]. The performance was increased in differentiating between women with malignant lesions and those with benign lesions or healthy women with normal mammograms. There is a similar study that could classify BI‐RADS category 4 lesions at an AUC of 0.9603 with a specificity of 95% and sensitivity of 88% using three plasma miRNA signatures (miR‐15a, miR‐101, and miR‐144) [41]. However, this was a single cohort study, and the findings have yet to be replicated.

There are more breast cancer studies related to miRNA signatures in differentiating between breast cancer patients and healthy women and those with benign breast lesions [7, 11, 42, 43]. However, there is a lack of a strong overlap of miRNA candidates among studies, which could be attributed to differences in data normalization and other factors or conditions, suggesting that methods of normalization may increase measurement variability, leading to misinterpretation of the measurements.

There are some limitations in this study. First, while the miRNA classifier performed well in determining the likelihood that a woman with a suspicious breast imaging finding will not have breast cancer or a pre‐cancerous diagnosis on a subsequent diagnostic biopsy, the sensitivity of the classifier for invasive breast cancer as compared to other subtypes such as DCIS warrants further investigation. Second, in general, women with benign lesions are followed according to standard practice guidelines. As we used a cross‐sectional study design to evaluate the miRNA classifier and long‐term follow‐up was beyond the scope, we did not perform follow‐up of any subject. Third, the number of subjects having 250 or more miRNA signals who were eligible to be included in the training and test sets was relatively small as compared to the 425 subjects tested. Although a sequencing approach such as next generation sequencing has an advantage in detecting low‐abundance miRNA in plasma/serum, variations in RNA extraction/purification and library preparation methods introduce sequencing bias and affect the miRNA profile detected [44, 45]. The microarray in this study is specially designed to detect miRNA without the use of any amplification procedure. This direct detection allows miRNA profiling to be more biologically relevant, unbiased, and accurate than those methods requiring amplification. However, this assay may result in a lower number of miRNAs detected. Therefore, in future studies, it will be crucial to minimize potential analytical confounding factors that may introduce bias in the performance of the miRNA classifier and to make the test more robust. Finally, the “rule‐out” performance of the miRNA classifier might be found to be improved in a larger prospective study and could be compared with that of existing diagnostic procedures to assess the potential advantage of incorporating the miRNA classifier into the standard of care workflow for the assessment of women with a suspicious breast imaging result (BI‐RADS Category 4 or 5).

## Author Contributions

**Hideo Akiyama:** conceptualization (lead), data curation (lead), formal analysis (lead), investigation (lead), methodology (lead), project administration (lead), validation (equal), writing – original draft (lead), writing – review and editing (equal). **Lora Barke:** conceptualization (equal), data curation (equal), investigation (equal), project administration (equal), supervision (equal), writing – review and editing (lead). **Therese B. Bevers:** conceptualization (equal), data curation (equal), investigation (equal), project administration (equal), supervision (equal), writing – review and editing (lead). **Suzanne J. Rose:** data curation (equal), investigation (equal), project administration (equal), supervision (equal), writing – review and editing (equal). **Jennifer J. Hu:** data curation (equal), investigation (equal), project administration (equal), supervision (equal), writing – review and editing (equal). **Kelly A. McAleese:** data curation (equal), investigation (equal), project administration (equal), supervision (equal). **Shellie S. Campos:** data curation (equal), investigation (equal), project administration (equal), supervision (equal). **Satoshi Kondou:** formal analysis (equal), methodology (equal), validation (equal), writing – original draft (supporting). **Jun Atsumi:** data curation (equal), formal analysis (equal), methodology (equal), validation (lead), writing – review and editing (supporting). **Thomas F. Soriano:** conceptualization (equal), data curation (lead), project administration (lead), writing – original draft (equal), writing – review and editing (equal).

## Conflicts of Interest

The authors declare no conflicts of interest.

## Data Availability

The data that support the findings of this study are available from the corresponding author upon reasonable request.
